# An evaluation of a multi-component adult weight management on referral intervention in a community setting

**DOI:** 10.1186/s13104-016-1901-1

**Published:** 2016-02-17

**Authors:** Kate Birnie, Lindsey Thomas, Clare Fleming, Sarah Phillips, Jonathan A. C. Sterne, Jenny L. Donovan, Julie Craig

**Affiliations:** School of Social and Community Medicine, University of Bristol, Bristol, UK; NHS South Gloucestershire, Bristol, UK; South Gloucestershire Council, Bristol, UK

**Keywords:** Obesity, Weight management, Diet, Physical activity, Behaviour change, Intervention

## Abstract

**Background:**

National Institute for Health and Care Excellence (NICE) guidance on adult weight management recommends interventions are multi-component. We aimed to assess the implementation and health benefits of a primary care referral to an adult multi-component weight management intervention in a community setting. The intervention was offered through Primary care in National Health Service (NHS) South Gloucestershire, UK, from Oct 2008 to Nov 2010, in partnership with statutory, community and commercial providers. The scheme offered 12 weeks’ community based concurrent support of dietary (Weight Watchers, WW), physical activity (Exercise on Prescription, EOP) and behavioural change (motivational interviewing) components to obese adults. Funding was available for 600 places.

**Results:**

Five hundred and fifty nine participants engaged with the intervention, mean age 48 years, 88 % female. Mean weight loss for all engagers was 3.7 kg (95 % confidence interval 3.4, 4.1). Participants completing the intervention achieved the largest weight reduction (mean loss 5.9 kg; 5.3, 6.6). Achievement of 5 % weight loss was higher in completers (58 %; 50, 65) compared to non-completers (19 %; 12, 26) and people who only participated in one commercial component of the intervention (either WW or EOP; 19 %; 13, 24).

**Conclusion:**

A multi-component weight management programme may be beneficial for weight loss, but a randomized controlled trial is needed to establish effectiveness and to evaluate cost.

## Background

The UK prevalence of adult obesity continues to be a major public health concern, almost a quarter of adults in England are now classified as obese [1] as defined by the World Health Organisation criterion of a body mass index (BMI) of 30 kilograms (kg)/metre (m)^2^ or above [2]. The impact on the individual can be considerable, as obesity is associated with a number of medical conditions [3], including diabetes [3–5], cardiovascular disease [3, 5] and cancer [3, 5, 6], and obesity is a major economic burden for the population [7, 8].

National Health Service (NHS) primary care is an obese patient’s first point of access to non-surgical, non-pharmacological weight management treatment. Currently at least 10 million UK adults are eligible for weight management interventions to reduce their risk of morbidity and mortality [1] and thousands of patients are identified daily through NHS initiatives for obesity, cardiovascular management and diabetes prevention. A primary route to weight loss is to achieve a negative energy balance (expending more calories than are consumed). This can be achieved by reducing dietary calorific intake or increasing physical activity or both. Diet and physical activity behaviours are both amenable to change and National Institute for Health and Care Excellence (NICE) guidelines state that multi-component lifestyle interventions that include behaviour change strategies to increase people’s physical activity levels and reduce dietary energy intake [9] are the treatment of choice.

We aimed to assess the implementation and potential health benefits of a novel multi-component weight management on referral intervention that integrated dietary, physical activity and behavioural change (including motivational interviewing) components in a in a community setting.

## Methods

### Study design

This study was a before and after evaluation of a multi-component weight management programme (“Weight management on referral”; WMOR) offered through NHS South Gloucestershire, UK, during the period from Oct 2008 to Nov 2010. Usual care prior to WMOR being available was general practitioner (GP) advice only.

### Recruitment of participants

Participants were referred into the scheme from 18 of 22 general practices that adopted the intervention in the South Gloucestershire region of England. Individuals were identified opportunistically by GPs and other health professionals in normal practice and referred to the programme if they met the inclusion criteria.

### Inclusion/exclusion criteria

Individuals were eligible for WMOR if they were: aged 18 years or over, if their BMI was 30 or above, or a BMI ≥ 28 with co-morbidities, had a ‘ready to change’ attitude (as assessed by the referring GP) and committed to complete both the physical activity and dietary elements of the intervention, and were comfortable in a group situation. People were excluded if they were currently (or in the last 3 months) on a commercial weight management programme, on anti-obesity drugs, on exercise on prescription, or pregnant.

### Sample size

Funding was available for 600 places.

### Intervention

The WMOR intervention included dietary, physical activity and behavioural change components; aiming to establish behaviour changes by building participants confidence and abilities to carry out exercise and make changes to diet. A commercial partner, Weight Watchers (WW) [10], provided participants with access to local, community-based, group WW meetings. Participants received free vouchers for 12 weekly supervised group sessions. At each meeting group members were weighed, there was a discussion led by the group leader and group members could share information and tips. A partnership with South Gloucestershire Council exercise on prescription team (EOP) [11] provided participants with access to local community-based leisure center gyms for one-to-one physical activity sessions. EOP practitioners are exercise specialists with comprehensive training in physical activity interventions, theory-led behaviour change techniques and dietary training and they delivered the motivational interviewing (MI) aspect of the intervention. MI is a directive focused non-judgemental person-centred counselling style that aims to work with resistance around behaviour change. This support aims to initiate behaviour changes, to strengthen and consolidate participants’ commitment and self-confidence to change. Individual goals are agreed and barriers to change, or to engaging with the intervention, are tackled.

The programme started with a 40 min individual session with baseline assessment, delivered by the EOP practitioner. Participants were helped to identify and set personalised realistic short term (12 week) and longer term goals for their weight and physical activity. The participants then attended 12 weekly sessions of separate dietary and physical activity components. All sessions were accessible via public transport. There was a motivational exit assessment conducted by an EOP practitioner at the end of the programme, where the EOP practitioner focused on helping participants become more self-directed and to build behaviour change into their daily lives.

### Ethics

This was a service evaluation; therefore ethical approval was not needed. We obtained written consent from patients to allow use, storage and transfer of personal data for NHS South Gloucestershire, Weight Watchers and South Gloucestershire Council.

### Measurements

Participants’ weight (kg) and BMI (weight [kg]/height [m]^2^) were measured objectively at baseline and at the weekly EOP and WW sessions. For this study, information on weight and BMI was used from the EOP sessions if available; otherwise recordings from WW were used. Final weight was the measurement taken at week 12, if available; otherwise the previous available measurement was used. Weight loss was calculated as the baseline weight minus the final weight; percentage weight loss was calculated as weight loss divided by baseline weight. BMI was classified according to the WHO definition of the degree of overweight or obesity in adults: 25–29.9 (overweight), 30–34.9 (obesity class 1), 35–39.9 (obesity class 2), ≥40 kg/m^2^ (obesity class 3). Physical activity levels were self-reported at baseline and at the weekly EOP sessions. Sedentary levels were considered to be less than 30 min of physical activity with moderate intensity per week and non-sedentary was ≥1 session of being physically active for 30 min per week. Postcodes of participants home addresses were linked to lower super output areas to match to South Gloucestershire deprivation quintiles from the index of multiple deprivation [12], ranging from 1 (least deprived) to 5 (most deprived). The domains used in the Indices of Deprivation 2010 were: income deprivation; employment deprivation; health deprivation and disability; education deprivation; crime deprivation; barriers to housing and services deprivation; and living environment deprivation.

### Statistical methods

All analyses were carried out using Stata version 12. Chi-square tests and analysis of variance were used to test for differences in baseline characteristics by WMOR completion status. An individual was considered to have completed the intervention if they attended 10 of the 12 sessions, for both EOP and WW. Final weight was compared across completion status subsets controlling for weight at start in linear regression; fully adjusted models controlled for age, sex, baseline activity levels and deprivation quintile. Logistic regression models were used to estimate odds ratios of achieving 5 % weight loss for different WMOR completion status subsets; fully adjusted models controlled for the same characteristics as in the linear regression.

## Results

### Participants

**Fig. 1:**
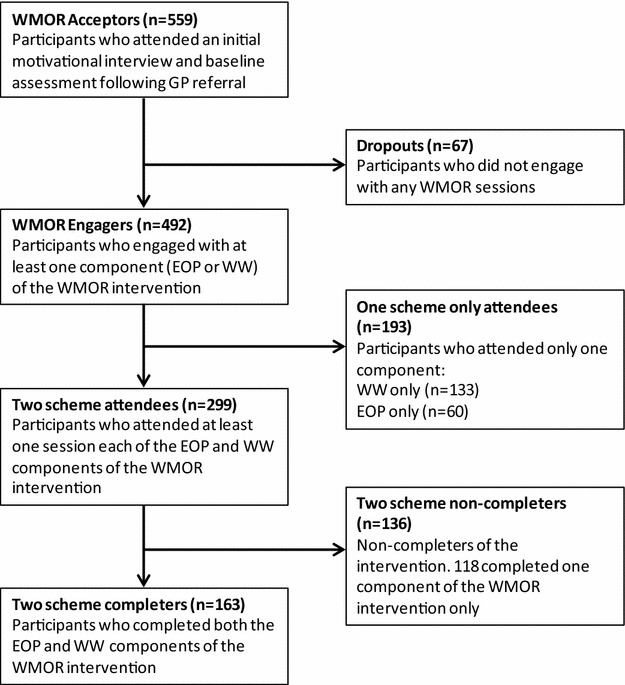
Flow of patients in the WMOR study

A total of 559 participants engaged with the WMOR intervention by attending the initial motivational assessment (Fig. 1). Of these, 67 participants (12 %) dropped out and did not subsequently engage with any WMOR sessions (‘dropouts’). Of the remaining 492 participants, 193 (39 %) did not follow the intended multi-component intervention and only attended one element (i.e., either WW or EOP, but not both; ‘one scheme only attenders’). There were 299 participants who started the intended intervention (i.e., attended both WW and EOP concurrently); of these, 163 (55 %) completed the intervention (‘two scheme completers’ and 136 (45 %) were non-completers of both the WW and EOP components (‘two scheme non-completers’). Of the non-completers, 118 (87 %) completed one component (either WW or EOP). The mean number of sessions attended across all participants who engaged with the scheme was 7 for both EOP and WW (Table 1).

**Table 1:** Characteristics of participants

	All WMOR acceptors	Two scheme completers	Two scheme non-completers	One scheme only attendees	Dropouts	P
Participants, N	559	163	136	193	67	
Age, years, mean (SD)	48 (14)	53 (15)	49 (13)	45 (13)	42 (12)	<0.001
Female, N (%)	494 (88 %)	144 (88 %)	120 (88 %)	173 (90 %)	57 (85 %)	0.735
Deprivation quintile^a^						
1 (least deprived)	96 (20 %)	38 (25 %)	19 (15 %)	31 (22 %)	8 (14 %)	
2	91 (19 %)	26 (17 %)	27 (22 %)	23 (17 %)	15 (26 %)	
3	96 (20 %)	34 (23 %)	21 (17 %)	32 (23 %)	9 (16 %)	
4	92 (20 %)	26 (17 %)	28 (23 %)	24 (17 %)	14 (24 %)	
5 (most deprived)	96 (20 %)	27 (18 %)	29 (23 %)	28 (20 %)	12 (21 %)	0.403
Missing data	88	12	12	55	9	
Baseline characteristics						
Weight, kg, mean (SD)	102 (19)	100 (18)	102 (21)	105 (19)	106 (20)	0.096
BMI, N (%)						
25–29.9	30 (5 %)	11 (7 %)	7 (5 %)	9 (5 %)	3 (5 %)	
30–34.9	181 (32 %)	60 (37 %)	48 (35 %)	55 (29 %)	18 (27 %)	
35–39.9	183 (33 %)	51 (31 %)	41 (30 %)	69 (36 %)	22 (33 %)	
40+	164 (29 %)	41 (25 %)	40 (29 %)	60 (31 %)	23 (35 %)	0.699
Sedentary physical						
Activity levels, N (%)	486 (95 %)	157 (97 %)	127 (95 %)	139 (93 %)	63 (95 %)	0.513
Mean sessions attended						
EOP, mean (range)	7 (0–12)	12 (11–12)	10 (1–12)	10 (2–12)^b^	0 (0–0)	<0.001
WW, mean (range)	7 (0–12)	12 (10–12)	7 (1–12)	6 (1–12)^b^	0 (0–0)	<0.001

^a^South Gloucestershire deprivation quintile

^b^For one-scheme only, calculated for those that attended each single scheme separately

The mean age of people recruited to the intervention was 48 (standard deviation [SD] 14) years (Table 1). Participants who completed the intervention were on average 53 years, those who dropped out of the intervention were younger, with a mean age of 42 years (p value for a difference between all subsets <0.001). Eighty eight percent of participants were female; there was no evidence of a gender difference between the completion subsets (p = 0.7). There was an equal distribution of participants from each of the local deprivation quintile areas. Average weight at baseline for all engagers was 102 (SD 19) kg. There was some evidence that baseline weight differed between groups (p = 0.096); the average weight for one scheme only attendees (105 kg) and dropouts (106 kg) was slightly higher than two scheme completers (100 kg) and two scheme non-completers (102 kg). At baseline, 95 % of participants were sedentary, there was no evidence that baseline physical activity differed between groups (p = 0.5).

### Weight loss

The mean weight loss for all engagers was 3.7 kg (95 % confidence interval [CI] 3.4, 4.1) (Table 2). The WMOR completers achieved the largest reduction in weight with a mean loss of 5.9 kg (95 % CI 5.3, 6.6), which is almost twice the mean weight loss of non-completers (mean loss 3.0 kg; 95 % CI 2.5, 3.4), or those that only participated in one element of the intervention (mean loss 2.4 kg; 95 % CI 1.8, 2.9). The average percentage weight loss for all engagers was 3.7 % (95 % CI 3.3, 4.0). Thirty two percent of all participants who engaged with WMOR achieved at least 5 % weight loss and 6 % of engagers achieved ≥10 % weight loss. Achievement of 5 % weight loss was higher in those that completed the intervention (58 % participants; 95 % CI 50, 65) compared to the non-completers (19 %, 95 % CI 12, 26) and one scheme only attendees (19 %, 95 % CI 13, 24).

**Table 2:** Weight loss achieved at 12 weeks

	N	Weight loss kg, Mean (95 % CI)	Mean percentage weight loss (95 % CI)	Percent achieving 5 % weight loss (95 % CI)	Percent achieving 10 % weight loss (95 % CI)
WMOR engagers	492	3.7 (3.4, 4.1)	3.7 % (3.3, 4.0)	32 % (28, 36)	6 % (4, 8)
Two scheme completers	163	5.9 (5.3, 6.6)	6.0 % (5.4, 6.6)	58 % (50, 65)	12 % (4, 17)
Two scheme non-completers	136	3.0 (2.5, 3.4)	2.9 % (2.4, 3.3)	19 % (12, 26)	0 %
One scheme only attendees	193	2.4 (1.8, 2.9)	2.3 % (1.8, 2.8)	19 % (13, 24)	4 % (1, 7)

### Comparison of weight loss outcomes by WMOR completion status

Two scheme non-completers were on average 3.06 kg (95 % CI 2.18, 3.95; p < 0.001) heavier at their final weight measurement than those who completed the WMOR intervention (Table 3). Two scheme non-completers had 84 % lower odds of achieving 5 % weight loss compared to two scheme completers (OR 0.16; 95 % CI 0.09, 0.28). Participants who only attended one element of the programme (EOP or WW) were on average 3.96 kg heavier (95 % CI 3.09, 4.83) at the end of the programme compared to two scheme completers and had 85 % lower odds of achieving 5 % weight loss (OR 0.15; 95 % CI 0.09, 0.27). Associations were not changed when controlling for age, sex, baseline physical activity and deprivation quintile.

**Table 3:** Final mean weight difference from linear regression and odds ratios for 5 % weight loss from logistic regression by WMOR completion subsets

	N	Mean weight difference^a^ in kg (95 % CI)	Odds ratio for 5 % weight loss^b^ (95 % CI)
Unadjusted models			
Two scheme completers	150	Ref (0)	Ref (1)
Two scheme non-completers	121	3.06 (2.18, 3.95)	0.16 (0.09, 0.28)
One scheme only attendees	129	3.96 (3.09, 4.83)	0.15 (0.09, 0.27)
P		<0.001	<0.001
Adjusted models^c^			
Two scheme completers	150	Ref (0)	Ref (1)
Two scheme non-completers	121	3.10 (2.20, 4.00)	0.15 (0.09, 0.27)
One scheme only attendees	129	4.05 (3.15, 4.94)	0.16 (0.09, 0.28)
P		<0.001	<0.001

^a^Linear regression model for weight at end, controlling for weight at start

^b^Logistic regression model for whether 5 % weight loss was achieved

^c^Models control for age, sex, baseline physical activity levels and deprivation quintile. Analyses were carried out on participants with complete data across these variables

### BMI change

**Fig. 2:**
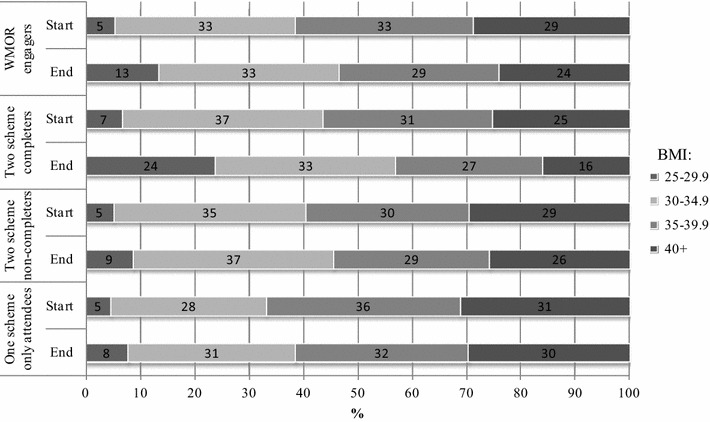
BMI classifications of WMOR participants at the start and end of the 12 week intervention

At baseline, 29 % of participants had a BMI in excess of 40 kg/m^2^ (obesity class 3) (Table 1). All groups show a shift of participants from higher to lower obesity classification from the start to the end of the 12 week intervention (Fig. 2). The highest beneficial change of obesity classification is shown by the WMOR two scheme completers, with a shift from 25 to 16 % in obesity class 3 by the end of the intervention, and an increase from 7 to 24 % in the overweight category (BMI 25–29.9, the healthiest obesity classification in the programme).

## Discussion

We performed an assessment of the implementation of a weight management intervention in practice. This aimed to contribute to the final development work before such an intervention could be tested in a randomized controlled trial (RCT). Over the 12 week period, 32 % of participants who engaged with the WMOR intervention and 58 % of those who completed the WMOR programme successfully achieved individual clinically important [13, 14] weight loss of ≥5 %. Meaningful beneficial shifts were seen in obesity classification for the population group in the programme. At baseline, 95 % participants were sedentary; participants who carried out the EOP element of the intervention will have increased their physical activity levels during the study period. If sustained, these increases may deliver further improvements in health, independent of effects of weight loss and support maintenance of the weight loss achieved for a longer time period [15]. Participants who completed the WMOR programme achieved better outcomes (greater weight loss and achievement of 5 % weight loss) compared to non-completers and one scheme only attendees (i.e., WW or EOP but not both).

The Lighten Up RCT reported the effectiveness for weight loss of a range of 12 week commercial or primary care led weight loss programmes compared with a minimal control of free activity vouchers [16]. Patients on commercial programmes were more successful in losing weight than those in NHS primary care and pharmacy programmes, at 12 weeks [mean difference 2.3 (1.3–3.4) kg] and the commercial provider used in our study, Weight Watchers, was the most successful of the providers examined. A service evaluation of three commercial providers of NHS primary care slimming on referral in North Somerset found that found WW provided a greater value for money than other providers with lower costs per kg lost, per percentage bodyweight lost and per BMI point change [17].

Outcomes in our study were similar to an audit of patients referred by the NHS to WW [18]; the average percentage weight loss for engagers in our study was 3.7 % compared to 3.1 %. Our study showed that clinically useful weight loss of 5 % was achieved by 32 % of engagers and the WW audit showed 33 % of all commenced referrals achieved a 5 % weight loss. The results for engagers from this study were slightly lower than two other non-randomized service evaluations comparing referral to commercial providers at 12 weeks [17, 19]. Our multi-component intervention was more intensive which may have led to the lower percentage of completers in the study, 33 % compared to 64 % [19] and 54 % [17]. As in our study, others have found that participants who completed the intervention achieved more weight loss than non-completers [17, 18]. When comparing outcomes for completers our results are comparable: NHS South Gloucestershire patients achieved a mean weight loss of 5.9 kg compared to 6.1 kg [19] and 6.4 kg [17]; and 58 % of completers achieved a 5 % weight loss compared to 61 % [17]. A key aspect to achieving health gains associated with dietary weight loss interventions is to sustain the weight lost and behaviour change achieved beyond 12 weeks. The Lighten Up RCT showed that a 12 week group based programme of weight management can also result in clinically beneficial amounts of weight loss that are sustained at one year [16]. However, obesity is a chronic, relapsing condition and the sustainability of weight loss achieved in short term interventions cannot be assumed. Around a third of participants who achieved a ≥5 % weight loss at the end of a 12 week commercial weight management programme regained weight within a year [16]. Hence, there is scope for improving outcomes from referral to primarily dietary focused lifestyle weight management interventions. Systematic reviews of RCTs have demonstrated that calorie restricted diets alone are more effective for weight loss than physical activity alone [20–22]. It has also been shown that dietary interventions can be enhanced: adding physical activity, particularly supervised physical activity, to dietary interventions increases weight loss by 2–3 kg [20, 23] at 1 year of follow up; adding established behaviour change techniques including motivational interviewing to weight loss interventions also increases their effectiveness [24, 25].

This study illustrates the potential benefits of implementing an adult multi-component weight management on referral intervention that includes dietary, physical activity and behavioural change elements, in a community setting. The programme targeted the initiation and establishment of behaviour change for participants. Limited primary care capacity and funding to treat obesity were the drivers for the study to use existing infrastructure based in the community to deliver the intervention components. We achieved a low cost, large capacity accessible intervention. Strengths of the intervention are that it was multi-component, as NICE guidelines recommend [9]. We are not aware of any other studies that have evaluated an intervention with these multiple components in a UK primary care population. The partnership working between PCT, GPs, local authority and Weight Watchers ensured the delivery approach of this integrated multi component service was truly patient centred.

Participants who completed all elements of the intervention achieved the most weight loss, but a major limitation is that the study was observational. It is plausible that completers were more motivated than non-completers, and weight loss differences between groups was due to baseline confounding rather than the effect of the intervention. Individuals were required to have a ‘ready to change’ attitude to be eligible for the intervention, therefore they may have achieved the observed weight loss over the study period without the intervention. The logical next step would be to carry out an RCT of the multi-component intervention. We do not have data on longer term outcomes of study participants, which would be useful to show whether weight loss is maintained, and further research is needed to assess the long-term clinical outcomes and the cost-effectiveness of referral to a multi-component intervention. An additional limitation was that when participants did not attend the exercise element of the intervention, we could not obtain weight measurements from the EOP sessions. In this case, we used measurements from WW sessions, these were made and recorded by WW group leaders and were not collected for the purpose of research. The intervention was only carried out in one region, South Gloucestershire, and may not be nationally representative. The participants had to have a ‘ready to change’ attitude, willing to complete both components and be comfortable in a group situation when they joined the programme, and so if this intervention were rolled out more broadly, response rates might be lower than seen here.

## Conclusions

We have demonstrated that it is possible to implement a multi-component adult weight management intervention in a community setting. The results from this study suggest the multi-component weight management programme may be beneficial for weight loss for obese individuals, but an RCT is needed to establish effectiveness and to evaluate cost.

## Abbreviations

BMI: body mass index
CI: confidence interval
EOP: exercise on prescription
GP: general practitioner
kg: kilogram
m: metre
MI: motivational interviewing
NHS: National Health Service
NICE: National Institute for Health and Care Excellence
RCT: randomized controlled trial
SD: standard deviation
WMOR: weight management on referral
WW: Weight Watchers
